# Between Scylla and Charybdis: thrombosis in children with hemophilia

**DOI:** 10.3389/fped.2023.1173549

**Published:** 2023-05-23

**Authors:** Jad El Maamari, Ali Amid, Marie-Claude Pelland-Marcotte, Soumitra Tole

**Affiliations:** ^1^ Division of Pediatric Hematology/Oncology, Department of Pediatrics, BC Children’s Hospital, Vancouver, BC, Canada; ^2^ Division of Pediatric Hematology-Oncology, CHU de Québec—Centre Mère-Enfant Soleil, Quebec City, QC, Canada; ^3^ Research Center of the CHU de Québec, Axe Reproduction, Santé de la Mère et de l’Enfant, Quebec City, Canada; ^4^ Department of Pediatrics, Schulich School of Medicine & Dentistry, Western University, London, ON, Canada; ^5^ Department of Pediatrics, Division of Hematology/Oncology, London Health Sciences Centre, London, ON, Canada

**Keywords:** hemophilia, thrombosis, children, emicizumab, CVAD

## Abstract

Thromboembolism is an infrequent complication in children with hemophilia that has been traditionally associated with the presence of a central venous access device. Novel rebalancing agents have shown promising results as prophylactic therapies to minimize the risk of bleeding but both thromboembolism and thrombotic microangiopathy have been reported as complications. The management of thrombosis in children with hemophilia is particularly challenging given the inherent risk of bleeding. In this paper, we present clinical vignettes to review the literature, highlight challenges, and describe our approach to managing thromboembolism in children with hemophilia.

## 1. Introduction

Hemophilia is an X-linked condition that results in the deficiency of factor VIII (FVIII) or factor IX (FIX) and can lead to life-threatening bleeding, hemarthrosis, and hemophilic arthropathy. The risk of bleeding is correlated with factor levels and is defined as mild (5%–40% factor level), moderate (1%–5% factor level) and severe (<1% factor level) (1). The use of prophylactic therapy to prevent bleeding complications is the cornerstone of hemophilia treatment in persons with severe hemophilia and those with moderate hemophilia with a bleeding phenotype. Frequent venipuncture can be painful and challenging in young children. Thus, a central venous access device (CVAD) is often needed for ease of administration and to institute optimal prophylaxis regimens at a young age. In the last few years, several non-factor replacement therapies have been developed to provide prophylaxis for persons with hemophilia. These novel agents are administered subcutaneously, making them substantially easier to administer in young children (2).

While it seems paradoxical that children with bleeding disorders would develop thrombosis, thrombotic complications are a rare but well-described complication in persons with hemophilia, especially with the use of CVAD. Clinical trials of non-replacement therapies have also reported thrombotic complications. However, there are no guidelines in the literature to guide the management of thrombotic events in children with hemophilia. In this article, we use clinical vignettes to present a review of the literature and our approach to the prevention and management of thrombotic complications in children with hemophilia. Of note, as gene therapy has not yet been studied in children, thrombosis related to gene therapy was outside the scope of this review.

## 2. Catheter-related thrombosis in children with hemophilia

**Case 1:** “A six-year-old male with severe hemophilia A presents with intermittent occlusion of his CVAD. He has a high-titre inhibitor and is currently receiving immune tolerance induction (ITI) with daily infusions of recombinant FVIII. His left-sided Port-A-Cath was put in place at the age of two to facilitate venous access in the setting of ITI. A compression ultrasound with Doppler is done to investigate the etiology of intermittent line occlusion and shows a non-occlusive thrombosis of the left internal jugular vein, at the tip of the CVAD. Is anticoagulation warranted for this child? Should the CVAD be removed?”

### 2.1. Use of CVAD in children with hemophilia

Persons with severe hemophilia require prophylactic administration of CFC to decrease the risk of bleeding and long-term consequences of arthropathy (3, 4). One of the challenging complications of CFC replacement therapy is the development of inhibitors or neutralizing antibodies (5). As inhibitors render CFC ineffective, they portend a very high risk of bleeding (6). Management of inhibitors in hemophilia generally involves intensive factor replacement therapy to induce tolerance (immune tolerance induction, ITI). ITI in children is particularly challenging because of the need of frequent, often daily, venous injections. The inability to ensure proper venous access may result in sub-optimal delivery of ITI and can have a tremendous negative effect on the quality of life of the child. CVADs have been shown to ensure timely, safe, and effective management of children with inhibitors (7). As such, the development of inhibitors in a young child typically results in the insertion of a CVAD. Even in young children without inhibitors, the use of a CVAD may facilitate effective delivery of prophylactic therapy. Blanchette et al. surveyed pediatric patients with hemophilia in North America and reported that approximately 80% of children on full dose prophylaxis therapy under the age of five required a CVAD (8). The installation of a CVAD comes with several risks such as perioperative bleeding, infection, thrombosis, and an increased need for inpatient admissions (9–11).

### 2.2. Epidemiology and risk factors of CVAD-associated thrombosis in children with hemophilia

Thromboembolism in persons with hemophilia is rarely reported in the literature probably due to the protective effect of hemophilia against blood clot formation. As such, data are scarce regarding their epidemiology and no established pediatric guidelines are available to guide treatment and follow-up.

One of the main causes of thrombus formation in children with a CVAD is endovascular injury (10). CVAD-specific characteristics such as the size, location, and type of catheter used may also contribute to the risk of thrombus formation (12). Persistently elevated endogenous levels of FVIII have been shown to increase the risk of TE in children and healthy individuals (13, 14). Thus, authors have hypothesized that frequent infusions of supratherapeutic doses of CFC may help contribute to the propagation of thrombosis once in place (15). Augustsson et al. described how the use of CFC can potentiate formation and propagation of thrombus through mechanisms that can be unrelated to tissue factor (16).

The incidence of thromboembolism in children with hemophilia who required a CVAD is highly variable in the literature, mainly due to variations in screening practices between institutions. In a systematic review and meta-analysis, Valentino et al. reviewed 2,973 cases of CVAD placement in 2,704 persons with hemophilia in different centers around the world. Fifty-five cases of thrombosis were identified, while 34.9% of the patients had CVAD placement for ITI. This review did not suggest a difference in rate of thrombosis in patients with and without factor inhibitors (17). Conversely, Van Dijk et al. noted that patients with hemophilia on ITI had higher rate of CVAD-related thrombosis (7.2 vs. 3.1/1,000 CVAD days), with 15% of all children with a CVAD in their cohort experiencing a thrombotic event (7). Journeycake et al. performed screening venography in 15 patients with hemophilia who had a CVAD for more than 12 months and reported eight (53%) patients with a DVT. All of the thromboses were found in patients with CVAD for more than 48 months (18). In the international immune tolerance study that surveyed 99 patients with 183 catheters, screening for asymptomatic thrombi was not performed and only one symptomatic DVT was identified (19). Carcao et al. hypothesized that continuation of frequent administration of factor replacement as part of ITI might cause a prothrombotic state, especially when the level of inhibitor is low, which might increase the risk of thrombus formation. The authors recommend monitoring of inhibitor titers and to stop frequent factor replacement when inhibitor titers drop below 2–3 Bethesda Unit (BU) (20).

### 2.3. Management of CVAD-related thrombosis in children with hemophilia

**Figure 1:**
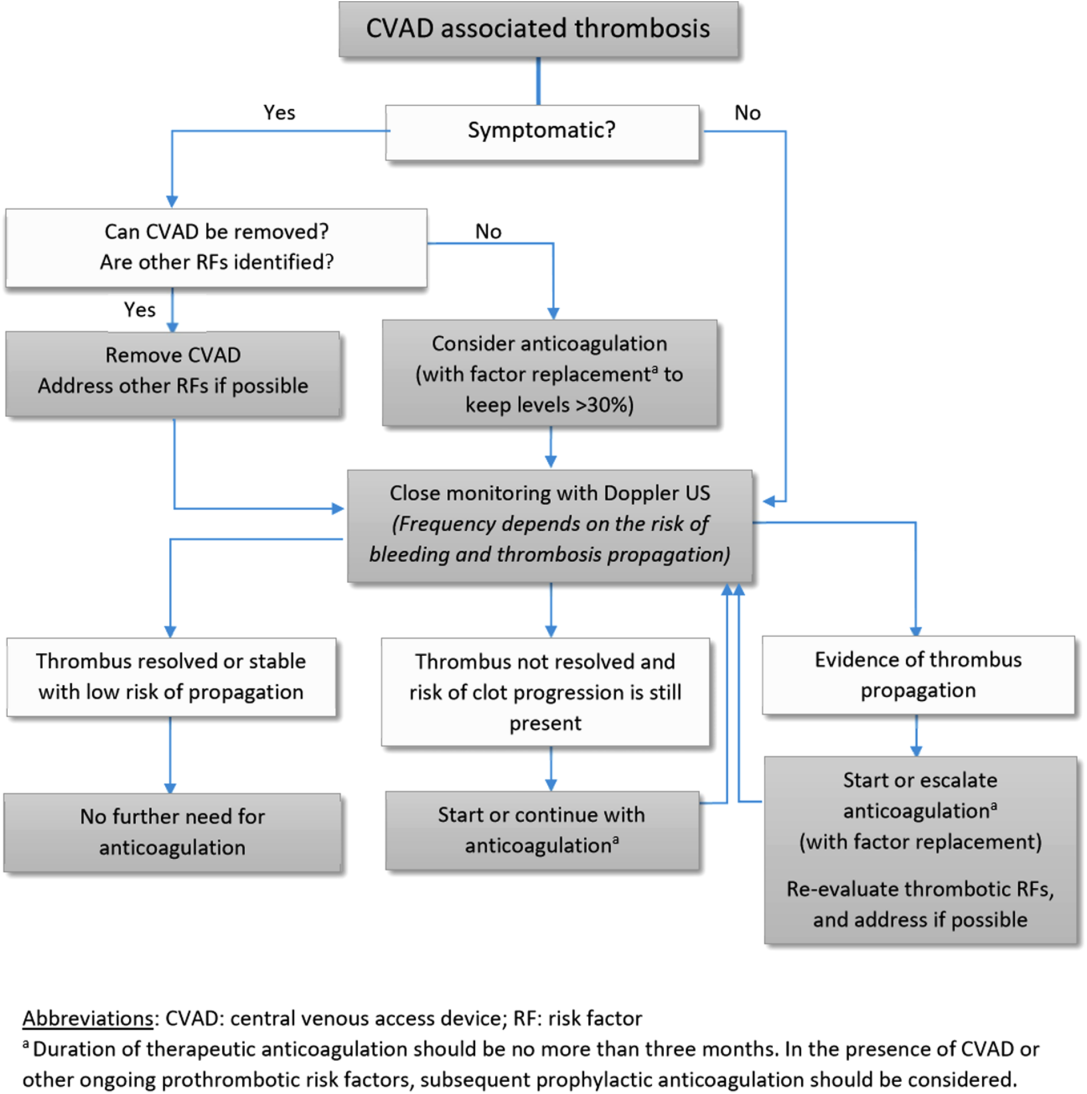
CVAD, central venous access device; RF, risk factor. ^a^Duration of therapeutic anticoagulation should be no more than three months. In the presence of CVAD or other ongoing prothrombotic risk factors, subsequent prophylactic anticoagulation should be considered.

Management of CVAD-related thrombosis is challenging in patients with hemophilia. The treatment approach should be individualized based on the patient's thrombotic and bleeding risk as well as the ongoing need for a CVAD; most commonly used modalities include expectative treatment, CVAD removal, and anticoagulation at either a prophylactic or a therapeutic dosing (Figure 1). The decision to initiate anticoagulation should be carefully made in consultation with hematologists. One report suggested removal of the CVAD with no anticoagulation as an efficacious method to prevent further propagation of the clot (18). In such situations, close monitoring with compression ultrasound is needed to ensure stabilization or degradation of the clot.

If anticoagulation is required, keeping the factor level above 30% might be required for safe anticoagulation (21) although this may be quite challenging or impossible in children with high-titer inhibitors. Dargaud et al. described the importance of balancing the use of anticoagulation and factor replacement, by aiming for therapeutic doses rather than reduced doses of anticoagulation in combination with CFC in such a way to have optimal anticoagulation activity around the factor plasma peak level (22). Alternatively, the use of Emicizumab (further described below) can be cautiously considered for prophylaxis while requiring anticoagulation. Weyand et al. reported a child with CVAD-related thrombosis in the context of bacteremia and ITI. The child was on prophylaxis with activated prothrombin complexes concentrates (aPCC) and developed significant enlargement of the thrombus, resulting in symptomatic obstruction of the right ventricular outflow tract. The child was transitioned to emicizumab and treated with low molecular weight heparin (LMWH) and received a total of six months of anticoagulation with removal of the CVAD at the three-month mark. A single hematoma at the emicizumab injection site was reported. Conversely, Barg et al. reported a child with a CVAD-related thrombosis treated with LMWH on emicizumab who experienced a fatal retroperitoneal bleed. As emicizumab results in thrombin generation of roughly 10%–15% FVIII-equivalent activity, emicizumab alone may be insufficient as prophylactic therapy while on therapeutic anticoagulation. Further data will be needed regarding the safety of anticoagulation while on emicizumab prophylaxis.

In persons with hemophilia who developed an inhibitor, given the high bleeding risk and the difficulty of achieving safe FVIII or FIX levels, one should be very careful before initiating anticoagulation. If the patient still necessitates a CVAD, anticoagulation with a lower therapeutic target, or using prophylactic dosing, might be considered to stabilize the clot and prevent propagation. Furthermore, existence of other risk factors for thrombosis propagation should be carefully evaluated for an informed decision.

When choosing anticoagulation, short-acting and reversible agents are recommended for patients with high risk of bleeding (21). In children, the most commonly used anticoagulants are unfractionated heparin and LMWH. While the use of direct oral anticoagulants (rivaroxaban and dabigatran) has recently been approved in children, there are no data on their use in persons with hemophilia and thus should be used with caution. The duration of anticoagulation should be tailored to the response to treatment and should be minimized to reduce risk of bleeding complication. We suggest performing a close follow-up ultrasound at six weeks, for possible discontinuation of anticoagulation should there be no thrombus propagation, based on the results of the KIDS-DOTT trial. Although not performed specifically in children with bleeding disorders, this randomized controlled trial has shown anticoagulation for 6 weeks to be non-inferior to a 3-month duration for thrombotic recurrence and clinically relevant bleeding for clearly provoked DVT when there is no residual occlusive thrombus at the 6-week radiological re-evaluation (23). Of note, this study excluded children with previous VTE, medical conditions putting the children at increased risk of recurrence, and high-risk thrombotic events (e.g., pulmonary embolism without DVT, more central PE, use or intent to use thrombolytic agents, etc.). If the risk factor for DVT is ongoing, anticoagulation therapy should continue in either therapeutic or prophylactic doses until the risk factor has resolved, based on the most recent pediatric guidelines for anticoagulation in children (24). Continuous assessment of the thrombotic and bleeding risk is crucial.

Thrombolysis may be indicated for life-threatening thromboembolism. For example, Carcao et al. reported a 10-year-old patient with severe hemophilia A presenting with superior vena cava syndrome necessitating mechanical thrombolysis. To minimize the risk of bleeding, his FVIII level was kept at >100% by frequent factor replacement alongside a continuous infusion of unfractionated heparin with a standard target heparin level between 0.3 and 0.7 IU/ml (20). The rationale of increasing the FVIII level to prevent major bleeding is supported by data published by analysis of 433 patients with moderate and mild hemophilia A. The study showed a decrease in bleeding episode by 18% for every 1% increase in factor VIII activity level (25).

In our case, we elected to remove the malfunctioning CVAD. As the patient was asymptomatic, and the thromboembolism was small and non-occlusive, no anticoagulation was used. Serial compression ultrasounds were performed to monitor for thromboembolism progression and showed thrombus stability. Peripheral intravenous injections were performed but ongoing difficulties with venous access prompted eventual placement of a second CVAD, with hemostatic coverage using bypassing agents. No secondary thromboprophylaxis was used.

## 3. Non-catheter related thrombosis in children with hemophilia

Thrombosis appears to happen very infrequently in children with hemophilia receiving CFC in the absence of a CVAD. Of note, a recent case series featured two children with mild hemophilia A (FVIII levels of 28% and 24%) who developed cerebral venous sinus thrombosis (CSVT) following head trauma. The first child had a concomitant subdural bleed and CSVT. She received intravenous FVIII replacement and, after ensuring stability of the intracranial bleed, LMWH for six weeks. Complete resolution of her subdural hematoma and cerebral venous sinus thrombosis was observed. The second case was initially treated with anticoagulation, which was discontinued due to an acute abdominal bleed. Regardless, she showed complete resolution of the CSVT. As shown by these examples, individualized management is required (26). In such cases, if possible, identification and elimination of any underlying modifiable risk factor for thrombosis is important.

## 4. Thrombosis associated with non-replacement therapies

**Case 2:** A three-year-old boy with severe hemophilia A with a high-titer inhibitor presents with a swollen right ankle after playing soccer with his siblings. He is on prophylaxis with subcutaneous emicizumab every 2 weeks. The emergency department physician calls for advice on hemostatic management. How should his bleed be treated?

### 4.1. Emicizumab

Non-factor replacement therapies are revolutionizing the prophylactic treatment of hemophilia. Emicizumab is a bi-specific monoclonal antibody which mimics the activity of factor VIII by bridging factor IXa and X. Hence, emicizumab accelerates FIXa induced factor X (FX) activation, which leads to thrombin generation (27, 28). Emicizumab provides a consistent, steady-state level of hemostasis, which has been approximated to a 10%–15% factor VIII activity level (29, 30). In an extensive clinical trial program, emicizumab has been demonstrated to significantly reduce annualized bleeding rates as compared to prophylaxis with bypassing agents in patients with inhibitors and FVIII prophylaxis in those without inhibitors (31–35). Emicizumab provides several potential benefits over traditional CFC replacement in children. It is administered subcutaneously every two to four weeks. Children, adolescents, and their caregivers have previously reported improvements in health-related quality of life outcomes (36). In children with inhibitors, there was a 99% reduction in annualized bleeding rates as compared to the use of bypassing agents, with 77% of children having zero bleeding events (32). As such, where available, emicizumab is quickly becoming the preferred prophylactic therapy for children with hemophilia A.

### 4.2. Management of bleeds for children with hemophilia receiving emicizumab

Despite the significant improvement in bleeding phenotype, children with hemophilia A on emicizumab can still experience breakthrough bleeding, especially in perioperative settings or after trauma. Management of bleeding in children on emicizumab requires personalised care based on the severity of bleed, bleeding phenotype of the patient, and the presence of an inhibitor.

In children without inhibitors, mild mucosal bleeding episodes may be treated with tranexamic acid alone. In moderate to severe bleeding, FVIII replacement should be given to target a FVIII level appropriate for the bleed severity. While FVIII has a binding affinity to FIXa and FX that is around 10 times higher than that of emicizumab (29). As such, FVIII preferentially binds to FIXa/FX when FVIII products are administered during a bleed. Several clinical trials have demonstrated the safety of concurrent FVIII therapy to treat bleeds or provide prophylaxis against surgery in children with hemophilia A, with no reported thrombotic events (33, 34, 37).

Special consideration should be given to the hemostatic management in children with hemophilia A with inhibitors. As with non-inhibitor patients, minor mucosal bleeding may be managed with tranexamic acid alone. FVIII replacement can be tried in patients with low-titers inhibitors (<5 BU), it is unlikely to be beneficial for patients with high-titer inhibitors. For moderate to severe bleeding bypassing therapy with aPCC or rFVIIa will be necessary (38–40). The Food and Drug Administration (FDA) has warranted a special warning for the use of aPCC products in patients receiving Emicizumab (41). As further explained below, emerging data pertaining to the synergistic prothrombotic effect of aPCC and emicizumab suggest that for the time being, rFVIIa should be prioritized to treat bleeds in these situations.

### 4.3. Thrombotic risk in persons with hemophilia receiving emicizumab

Multiple concerns of increased risk of thrombotic microangiopathy (TMA) and thromboembolism were reported in the literature with emicizumab (Table 1). In the HAVEN-1 clinical trial of emicizumab prophylaxis in adolescents and adults with hemophilia A with inhibitors, the development of thrombotic microangiopathy (TMA) was reported with the concurrent use of emicizumab and aPCC (31–34). Subsequently, out of 8 patients treated with aPCC (>100 U/Kg/24 h) for more than 24 h, 3 patients developed TMA. Two of the cases resolved while one individual experienced fatal rectal hemorrhage. An additional case of TMA was reported post FDA approval, that resolved with cessation of aPCC use (52). In addition, two cases of thromboembolism were also reported with the concurrent use of emicizumab and aPCC. These adverse events resulted in a FDA “black box” warning for TMA or thromboembolism when emicizumab and aPCC are used concomitantly. Nissen et al. reported 3 cases of TE and 0 cases of TMA in 985 patients on emicizumab. All 3 cases of thromboembolism occurred on emicizumab monotherapy, but patients had multiple other risk factors (48). To date, we are not aware of pediatric cases of TMA associated with emicizumab use, which may be explained by the “black box” warning being issued prior to pediatric approval rather than developmental differences in coagulation in children.

**Table 1:** Thrombotic adverse events related to emicizumab.

Reference	Study design	Population		Sample size	Thrombotic adverse events^a^
		Age (years)	Inhibitors		
Oldenburg, 2017 HAVEN-1 (31)	Open-label RCT	12–75	Yes	109	TMA associated with concomitant aPCC (*n* = 3) TE associated with concomitant aPCC (*n* = 2): cavernous sinus thrombosis; skin necrosis-superficial thrombosis TE (*n* = 1): device occlusion of a PICC line
Young, 2019 HAVEN-2 (32)	Non randomized study	1–15	Yes	88	None
Mahlangu, 2018 HAVEN-3 (33)	Open-label RCT	≥ 12	No	152	None during the study; TE (*n* = 1): myocardial infarction during follow-up
Pipe, 2019 HAVEN-4 (34)	Non randomized study	12–65	Both	48	None
Shima, 2019 HOHOEMI (37)	Non randomized study	≤12	Both	13	None
McCary, 2020 (42)	Observational study	<55 (0.16–55)	Both	93	None
Shang, 2020 (43)	Registry data (EUHASS Database)	NR	Both	148	TE associated with concomitant aPCC (*n* = 1): myocardial infarction
Barg, 2021 (44)	Observational study	1–56	Both	107	TE (*n* = 1): CVAD-related thrombosis
Howard, 2021 (45)	Registry data (Roche Global Safety Database)	NR	Both	NR	TMA associated with concomitant aPCC (*n* = 4) TE associated with concomitant aPCC (*n* = 2) TE non-associated with concomitant aPCC (*n* = 37): CVAD-related TE (*n* = 7)
Dubé, 2022 (46)	Observational study	0–55	Yes	17	NR
Jimenez-Yuste, 2022 STASEY (47)	Non randomized study	≥12	Yes	195	TE (*n* = 2): myocardial infarction; post-operative localized thrombus at site of tooth extraction
Nissen, 2022 (48)	Registry data (EUHASS Database)	NR	Both	895	None
Yang, 2022 HAVEN 5 (49)	RCT	≥ 12	Both	70	None
Maria, 2023 (50)	Registry data (EUDRA Vigilance database)	NR	NR	NR	TMA (*n* = 1) TE (*n* = 22): Arterial TE (*n* = 14); Venous TE (*n* = 8) Disseminated intravascular coagulation (*n* = 1)
Négrier, 2023 HAVEN-6 (51)	Non randomised study	12–36	No	72	TE (*n* = 1): grade 1 thrombosed hemorrhoids

RCT, randomized controlled trial; NR, not reported TMA, Thrombotic microangiopathy; TE, thromboembolism; CVAD, central venous access device; aPCC, activated prothrombin complex concentrates; PICC, peripherally inserted central catheter.

^a^ Some TE or TMA events could be counted twice, as events captured from post-marketing databases may have been reported separately elsewhere.

^b^ Adult refers to adolescents and adult population (cut-off age typically ≥12 years old).

While emicizumab mimics the action of FVIII by binding the activated FIXa (enzyme) to the FX (substrate), several notable differences exist in its mechanism of action that likely contribute to its thrombotic potential. Compared to FVIII, emicizumab has a low affinity for the enzyme and its substrate, and does not distinguish between the zymogen and the enzyme (e.g., FIX vs. FXIa and FX vs. FXa) (29, 53). In addition to bridging FIXa and FX, FVIII promotes phospholipid binding and stabilizes the FIXa active site. As emicizumab cannot complete these additional actions, its action is not limited to the platelet cell surface and excessive thrombin generation may occur anywhere in the vasculature, including in the endothelium. Finally, the action of FVIII is highly regulated, which is not the case with emicizumab, which leads to a persistent, low-grade activation of the coagulation system (53). When aPCC is administered to a patient receiving emicizumab, this leads to a prolonged activation of procoagulant pathways by providing large amounts of FIX (and of FIXa, to a lesser extent) (29, 54). In a seminal study, Hartmann and colleagues have shown that the *in vitro* combination of a sequence-identical analog of emicizumab and aPCC leads to a 17-fold, synergistic increase of thrombin generation (54).

Recently, Kizilocak et al. reported the *in vitro* and *in vivo* effect of escalating doses of aPCC in nine persons with hemophilia with inhibitors, aged 5–27 years old, on emicizumab. While excessive thrombin generation was seen *in vitro* with standard dosing of aPCC, most patients had normal thrombin generation *in vivo* with doses of aPCC up to 75 units/kg, suggesting that low doses of aPCC may be considered in specific circumstances (55). Importantly, suboptimal *in vivo* endogenous thrombin generation was noted with subtherapeutic doses of aPCC.

In addition to TMA, there have been several reported episodes of thromboembolism associated with the use of Emicizumab (Table 1). As noted above, during the HAVEN-1 clinical trial two cases of thromboembolism occurred with concurrent aPCC use, which included one case each of cavernous sinus thrombosis and superficial thrombophlebitis. Both cases were managed with discontinuation of aPCC without the need for anticoagulation. Emicizumab was restarted in one patient without any further thromboembolism recorded (31). Importantly, no thrombotic events were reported in children in the HAVEN clinical trial program, nor the HOHOEMI single arm study of prophylaxis in young children (37). However, a real-world study of emicizumab reported an infant with a CVAD-related thrombosis without concurrent use of bypassing agent therapy. Unfortunately, this child had a fatal bleed while on concurrent anticoagulation therapy with LMWH (44).

Levy et al. analysed data from HAVEN 1–4 trials and reported no cases of TMA or TE with the concomitant use of emicizumab and rFVIIa (56). A recent publication reported an adult with hemophilia A and with inhibitor on emicizumab who received rFVIIa to treat an ankle bleed and experienced a mycocardial infarction and pulmonary embolism. However, the patient had multiple comorbidities that may have contributed the thrombotic event (57).

While not related to acute bleed management, an important consideration in assessing thrombotic risk is the use of frequent, high-dose FVIII replacement as part of ITI while on emicizumab prophylaxis, often times using a CVAD. Batsuli and colleagues reported the “Atlanta” protocol with the concurrent use of emicizumab and ITI with no thrombotic events noted, while the results were promising for inhibitor eradication (58). The ongoing MOTIVATE trial (ClinicalTrials.gov Identifier: NCT04023019) is investigating the safety of this approach prospectively.

Based on these observations, rFVIIa (eptacog alfa) is the treatment of choice in children with hemophilia A with inhibitors who experience breakthrough bleeding or require surgery while on emicizumab. The National Hemophilia Foundation recommends the use of standard initial dosing of rFVIIa at 90–120 mcg/kg at no more than a q2 h interval. Alternatively, in adolescents older than 12 years of age and adults, an alternate rFVIIa product, ectacog beta, may be used. The recommended dosing is 75 mcg/kg at no more than q3 h interval. While duration of therapy is individualized, most bleeding events are expected to resolve with 2–3 doses of rFVIIa (59). In situations where rFVIIa is not available or if the bleed is non-responsive to rFVIIa, low dose aPCC can be used, using doses up to 50 units/kg with a maximal daily dose of 100 units/kg (60). Frequent blood work to monitor for TMA should be performed, including twice-daily complete blood count, reticulocyte count, blood smear looking for schistocytes, bilirubin, haptoglobin, creatinine, lactate dehydrogenase, and d-dimers (61). Should TMA develop, the recommendation is to stop aPCC and monitor the patient closely. Most of the published cases reported resolution of TMA with discontinuation of aPCC and supportive care, and occasionally plasmapheresis (31).

For our case, we opted to treat the ankle hemarthrosis with rFVIIa (eptacog alfa) with initial dose of 90 mcg/kg. The child required a second dose to achieve satisfactory hemostasis and was admitted for observation. Given the availability and response to rFVIIa, the use of low dose aPCC was not considered. He was evaluated by physiotherapy and given recommendations around weight-bearing and stretching. The benefits of inhibitor eradication, namely the ability to use FVIII to treat bleeds in tolerized children was re-discussed with the family and initiation of ITI was planned.

## 5. Rebalancing agents

Other promising therapeutic agents for patients with severe factor deficiencies include rebalancing agents, targeting natural anticoagulants. This concept stems from “rebalanced coagulation” observed with liver disease, in which levels of procoagulants and anticoagulants are similarly reduced, such that overall thrombin generation remains near normal (53). Additionally, it was recognized that co-inheritance of a thrombophilic trait along with hemophilia led to a milder bleeding phenotype. Several products are currently in development, and none are currently approved and marketed. Fitusiran is a small interfering RNA (siRNA) that silences post-transcriptional hepatic expression of the *SERPINC1* gene, thereby reducing antithrombin levels in a dose-dependent manner. Concizumab and Marstacimab are humanized monoclonal antibodies targeting the Tissue Factor Pathway Inhibitor (TFPI) binding site for FXa. Table 2 summarizes thrombotic complications encountered in clinical trials with rebalancing agents. Considering the little combined experience using these agents and the scarcity of data, caution and monitoring when providing procoagulant therapy for surgery or breakthrough bleeds will be required when using these products. This is especially true for young children, given the well-known concept of developmental hemostasis (69) and its implication of the fine balance between bleeding and thrombosis.

**Table 2:** Thrombotic adverse events related to non-factor replacement in trials.

Non-factor therapy	Age (years)	Mechanism of action	Reported thrombotic events
Fitusiran	≥12	Rebalancing agent RNAi against AT	Fatal cerebral sinus venous thrombosis with concurrent use of high-dose FVIII concentrate. Further thrombotic events on study leading to refinement of target AT levels (53, 62–64)
Concizumab	≥18	Rebalancing agent Monoclonal antibody against TFPI	TE: 5 episodes in 3 patients, all occurring with the use of concomitant hemostatic medications. (65, 66) *Trials temporarily suspended and restarted with risk mitigation strategy
BAY 1093884	≥18	Rebalancing agent Monoclonal antibody against TFPI	TE: 3 episodes in 24 patients in phase 2 trial leading to termination of clinical trial program (67).
Marstacimab	≥19	Rebalancing agent Monoclonal antibody against TFPI	No reported TE events (68)

TFPI, tissue factor pathway inhibitor; AT, antithrombin; FVIII, factor VIII; TE, thromboembolism.

## 6. Conclusion

Despite being at high risk of bleeding, children with hemophilia can still develop complications related to thrombosis. The two presented cases reflect the most frequent (CVAD-related) as well as novel (TE risk with rebalancing agents) causes of TE encountered in children with hemophilia A. We advocate for an individualized approach to management with balancing the risks of bleeding and clotting. When opting not to treat TE in children with hemophilia, close radiological surveillance for thrombus progression is suggested. The development of several novel rebalancing agents as prophylactic therapy for persons with severe hemophilia A bring the promise significant improvements in bleeding and, most importantly for children, the possibility of subcutaneous administration. Consequently, the uptake of such therapies may result in a marked decrease in the use of CVADs, the biggest risk factor for TE in children. At this time, however, there remains an incomplete understanding of the thrombotic risks of rebalancing agents, and how to effectively use concomitant hemostatic therapy when there is breakthrough bleeding. In the future, it will be vital to have real-world studies assessing the long-term risks of not only bleeding and arthropathy, but also TE in children with hemophilia.
